# Acromioclavicular and coracoclavicular ligamentous insertion distances depend on the scapular tilt and decrease with anterior direction of the inferior scapula angle

**DOI:** 10.1007/s00167-022-07126-9

**Published:** 2022-09-02

**Authors:** Julia Sußiek, Jens Wermers, Michael J. Raschke, Elmar Herbst, Felix Dyrna, Oliver Riesenbeck, J. Christoph Katthagen

**Affiliations:** 1 Department of Trauma-, Hand- and Reconstructive Surgery, University Hospital Münster, Waldeyerstr. 1, 48149 Münster, Germany; 2 Gelenkzentrum Rose, Richard-Lehmann-Str.21, 04275 Leipzig, Germany

**Keywords:** Acromioclavicular joint injuries, Biomechanics, Shoulder, Coracoclavicular ligaments

## Abstract

**Purpose:**

A variety of reconstruction techniques exist for the operative treatment of a ruptured acromioclavicular and coracoclavicular ligamentous complex. However, the complication rate remains high; between 5 and 89%. The intraoperative distance between the clavicle, acromion and coracoid is important for the refixation quality. In this study, the influence of scapular deflection on coracoclavicular and acromioclavicular distances was analysed.

**Methods:**

The ligamentous insertions of 24 fresh-frozen human scapulae were exposed. The coracoclavicular and acromioclavicular ligaments were referenced and captured in a rigid body system using a three-dimensional (3D) measurement arm. The inferior angle of the scapula was manually pulled into maximum anterior and posterior deflection, simulating a patient positioning with or without dorsal scapular support, respectively. Based on the rigid body system, the distances between the ligamentous insertions were calculated. Statistical evaluation was performed by setting the distances in anterior deflection to 100% and considering the other distances relative to this position.

**Results:**

The scapular deflection had a considerable impact on the distance between the ligamentous insertions. Concerning the conoid ligament, the mean distance was almost doubled when the inferior angle pointed posteriorly compared to anterior deflection (195.3 vs 100.0%; *p* = 0.028). The insertion of the acromioclavicular capsule also showed a significant association with the direction of deflection (posterior = 116.1% vs. anterior = 100%; *p* = 0.008).

**Conclusion:**

Dorsal support shifting the inferior angle of the scapula anteriorly reduces the distance between the ligamentous insertions. Therefore, a patient position on a shoulder table with posterior support of the scapula is recommended to reliability reduce the acromioclavicular joint.

## Introduction

Acromioclavicular (AC) injuries represent up to 11% of shoulder girdle injuries [19]. In the majority of cases, the injury mechanism is a fall on the adducted arm [14]. The AC joint dislocation can be classified according to Rockwood [9]. Most of the Rockwood type IV, V and VI AC joint injuries need to be reduced and stabilized surgically [1, 6, 7, 17]. For the surgical treatment of acute AC joint injuries, there is widespread consensus to perform an arthroscopic anatomic reconstruction with a suspensory device [18]. Most of these arthroscopic procedures are performed in a beach chair position. In this position, the scapular deflection is often ‘free-floating’ without dorsal support [10].

Despite the varying treatment strategies, the complication rate remains high and varies between 5 and 89% [5]. A common complication after operative treatment is loss of reduction. In a prospective multicentre study, Clavert et al. reported an incidence of 41.3% for radiological failure, when the side-to-side difference of the coracoclavicular distance was more than 50% increased during follow-up. This radiological failure was mirrored by a lower Constant Score and higher DASH score [4]. Marsalli et al. noted recently a loss of reduction ≥ 1 mm in 78% of their patient cohort, whereby a loss of reduction > 5 mm increased the risk to develop pain in the AC joint [12]. Other studies report a clinical loss of reduction rate of 12.5–14% [3, 16]. Chen et al. identified the coracoid EndoButton position, weight-bearing time of the upper limb and osteolysis as potential risk factors for loss of reduction in their study cohort [3].

Until today, several studies investigated the anatomical length of coracoclavicular and acromioclavicular ligaments in a fixed position. In these studies, length variations were analysed under stable conditions of the shoulder girdle. The length variations in dependence on the scapular position relative to the clavicle were not yet analysed in detail. However, scapular support during operative treatment may be an important factor concerning ligamentous reconstruction. Thus, the aim of this study was to evaluate a potential anatomic-mechanic risk factor for the loss of reduction—the scapular position during the surgical procedure. Intraoperatively, the orientation of the lateral clavicle in relation to the acromion is often used as a control for proper AC-reduction. Thereby the position of the acromion and the coracoid may depend on the patient’s scapular orientation. As the reconstruction quality can be improved and a good functional outcome can be achieved with an overreduction [11, 13], it was assumed that higher stability can be achieved when the ligamentous insertions are as close as possible to each other during the surgical treatment.

Until today the length of the coracoclavicular and acromioclavicular ligaments was analysed in a fixed position. As the scapula position may vary intraoperatively, the aim of this study was so to investigate the length variation in relation to different positions of the scapula. As a previous study proved that the coracoclavicular ligaments insert on the superior surface of the coracoid and the inferior surface of the clavicle [2], it was hypothesized that the ligamentous insertions are closer to each other when the inferior angle points anteriorly, such as when the patient is operated on a shoulder table with dorsal support.

## Material and methods

In this biomechanical study, 24 fresh-frozen human scapulae (9 male, 15 female) were investigated. The mean age was 80.7 years (SD ± 7.4). During their lifetime, all donors provided written consent to contribute their bodies for research purposes (IRB No. 2014-421-f-N, Institutional review board of the Medical Association of Westphalia-Lippe and the University of Münster).

### Specimen preparation

The fresh-frozen specimens were thawed at room temperature. All superficial soft tissue was dissected and the conoid and trapezoid ligaments as well as the acromioclavicular capsule were exposed. The medial end of the clavicle was embedded in a squared frame with polyurethan (PU) casting (Rencast, Gößl + Pfaff, Karlkron/Brautlach, Germany) and rigidly mounted in a bench vice to ensure a static position of the clavicle while the scapula was ‘free-floating’.

### Test set-up

**Fig. 1:**
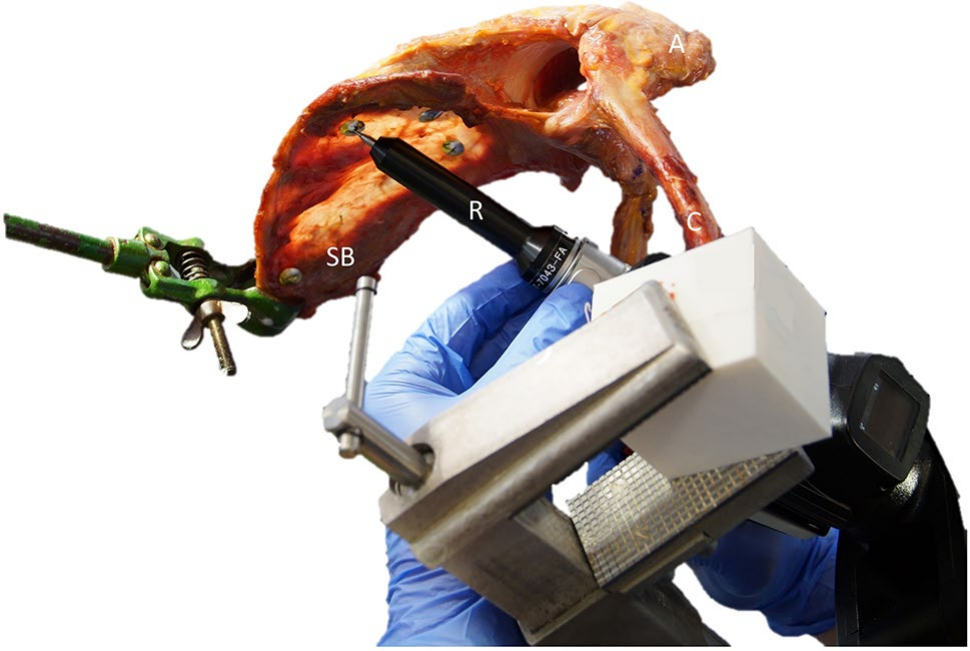
Fixation of the scapula (SB) and clavicle (C) during initial measurements of anatomical ligamentous insertions and reference pins for the definition of a rigid body system. (*R* Romer arm, *A* Acromion)

All measurements were performed by two investigators. First, the scapula and clavicle were fixed in a stable position with custom-made clamps to avoid any movement during the initial measurements of ligamentous insertions and the referencing of a rigid body system [2]. The three-dimensional (3D), anatomical measurements were performed using a tactile 3D measuring arm (Absolute Arm 8320-70, Hexagon, Stockholm, Sweden) with a measurement accuracy of less than 0.05 mm. With the measuring arm, the insertions of the conoid and trapezoid ligaments at the scapula and clavicle as well as the AC capsule were captured at defined anatomic landmarks. Furthermore, four rigid reference pins were placed spread over the scapular body (Fig. 1). These were also captured with the measuring arm in a fixed scapular position to create a transformation between anatomical landmarks and reference pins. The distances between the anatomic landmarks were calculated afterwards based on the initial measurements and the rigid body system. For each specimen, the selected landmarks were verified by two investigators.

**Fig. 2:**
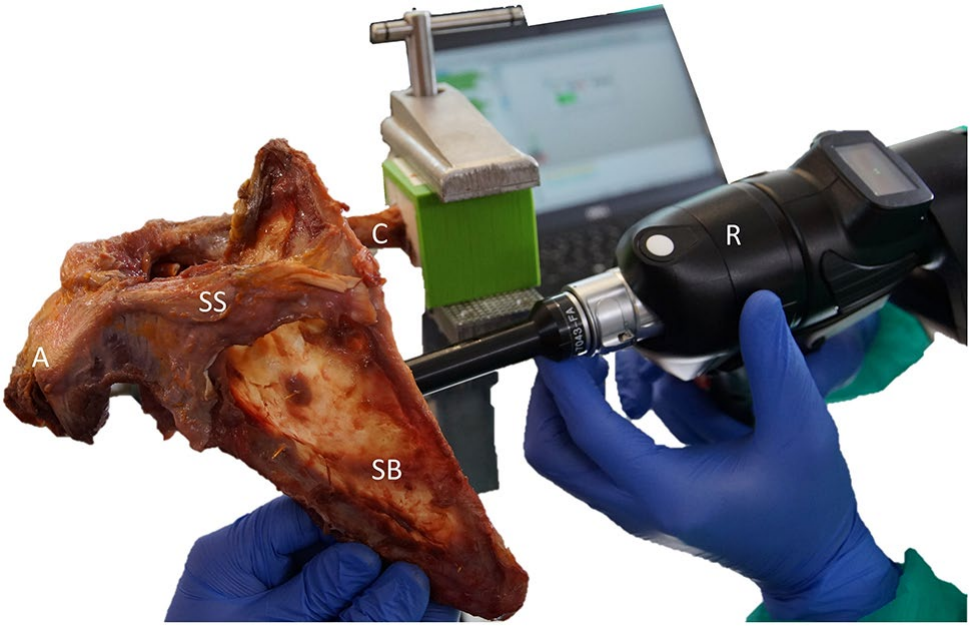
Deflection of the inferior angle into maximum anterior, posterior, medial and lateral position. Each position was repeated and captured three times by two different assistants, resulting in six distance measurements for each ligamentous insertion and scapular deflection. (*A* Acromion, *SS* Scapula spine, *SB* Scapula body, *C* Clavicle, *R* Romer arm)

After the initial measurements, the clamp fixing the scapula was detached, so that the scapula was free-floating. For the determination of distances, the inferior angle of the scapula was manually pulled to its maximal deflection in anterior—posterior and medial—lateral direction (Fig. 2). By doing so, the deflection in anterior direction was representing a patient positioned in a shoulder table with dorsal support. Posterior deflection simulated a freely hanging position without any dorsal support of the shoulder. The pins in the scapular body were captured with the measuring arm in each maximum deflection. Due to the static clavicle and the initial measurements with a rigid body definition of the scapula, the distance between ligamentous insertions could be precisely calculated. For each scapular position, the deflection and acquisition of reference pins was repeated three times. Afterwards, a second person moved the inferior angle of the scapula and the measurements were repeated three times for each position, resulting in a total of six distance measurements for each ligamentous insertion and scapular deflection.

### Statistical analysis

A customized MATLAB Script (MATLAB 2019b, MA, USA) was used for data analysis. Based on the rigid body system, the distances between the insertions of the conoid and trapezoid ligament and the AC capsule were calculated in each deflection (anterior, posterior, medial and lateral). The mean distance was then calculated from the six measurements for each specimen in each position. The length variation was reported in percent, whereby the distance in the maximum anterior deflection was set to 100%.

A post-hoc power analysis was performed using G*Power 3.1.9.4 (Franz Faul, Edgar Erdfelder, Albert-Georg Lang, and Axel Buchner). Based on the data of the current study and the number of specimens used the effect size was 0.41 (anterior–posterior deflection of the scapula) and 0.64 (medial–lateral deflection of the scapula) with a corresponding power of 0.98 (anterior–posterior) and 0.99 (medial–lateral) for the conoid ligament. For the analyses of the trapezoid ligament, the effect size was 0.43 (anterior–posterior) and 0.36 (medial–lateral) with a resultant power of 0.99 (anterior–posterior) and 0.95 (medial–lateral).

The statistical analysis was performed with GraphPad Prism (GraphPad 8.3.1, San Diego, USA). The results were compared with a Repeated Measures one-way ANOVA followed by a Tukey’s test with a correction for multiple comparisons. The level of significance was set to *p* < 0.05.

## Results

**Fig. 3:**
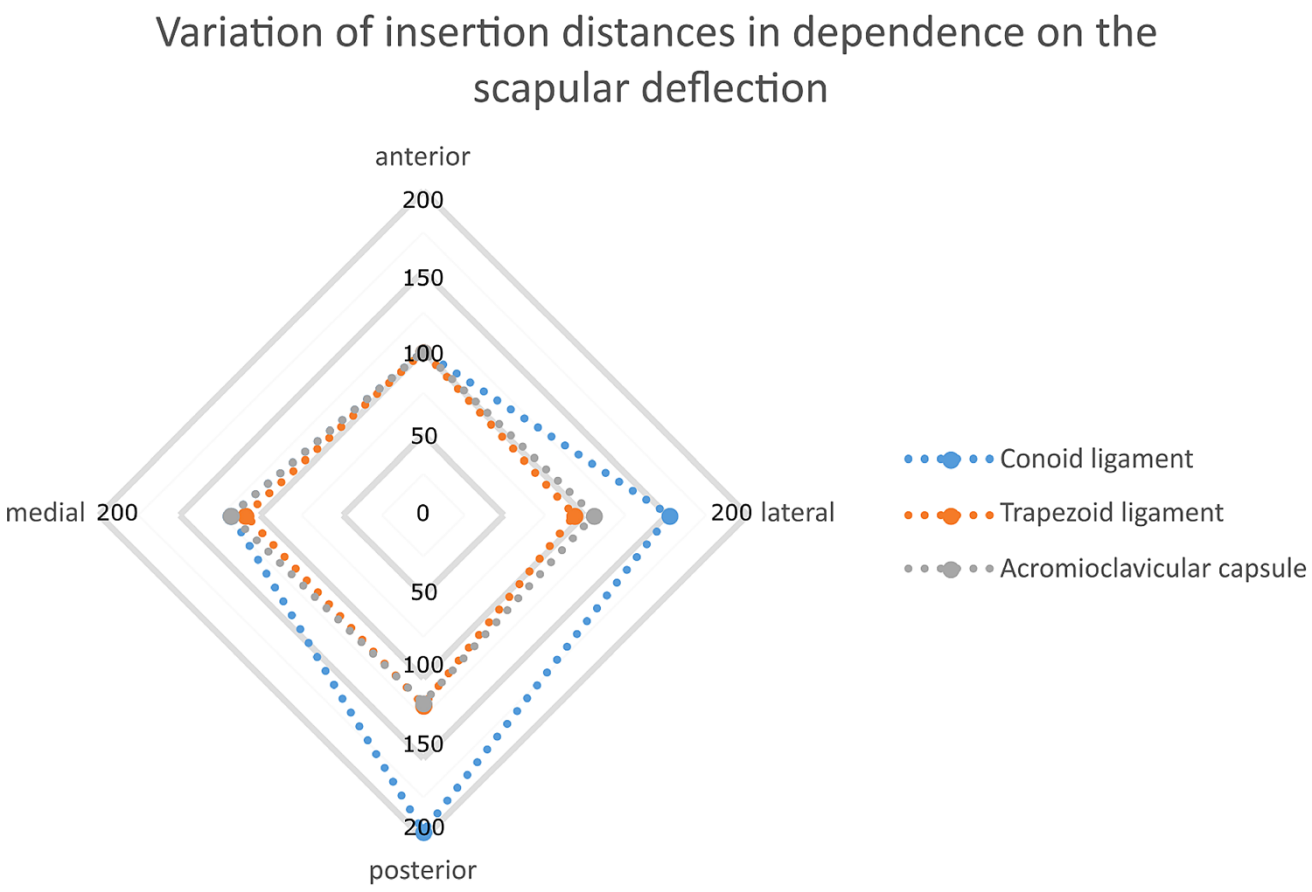
Variation of insertion distances in [%] relative to anterior deflection (100%), dependent on the deflection of the inferior angle of the scapula. The largest distance between the tendinous insertions of CC ligaments was calculated when the inferior angle pointed in posterior direction, representing a scapula without any dorsal support. For the AC capsule, medial and posterior deflection resulted in larger distances than lateral or anterior deflection. The scapular deflection had a higher impact on the ligamentous insertion distances for the conoid ligament than for the trapezoid ligament.

The insertions of the conoid ligament were significantly more apart from each other when the inferior angle of the scapula was deflected posteriorly (mean distance changes: posterior = 195.3% (SD ± 154.1%) vs. anterior = 100.0% (SD ± 0.0%), *p* = 0.028). Comparing the deflection in medial and lateral directions, the ligamentous attachments were significantly closer when the inferior angle was orientated medially [mean distance changes: medial = 118.5% (SD ± 76.3%) and lateral = 151.5% (SD ± 69.9%), *p* = 0.001] (Fig. 3).

A similar pattern was seen for the trapezoid ligament. With the inferior angle in posterior deflection, the ligamentous insertions were slightly more apart, but the difference was not significant [mean distance changes: posterior = 117.0% (SD ± 39.0%) vs. anterior = 100.0% (SD ± 0.0%), *p* = 0.174]. However, when the inferior scapula pointed laterally, the attachments were closer in comparison to medial deflection [mean distance changes: medial = 109.2% (SD ± 20.9%) vs. lateral = 94.0% (SD ± 22.7%), *p* = 0.001].

The insertions of the AC capsule were significantly closer when the inferior angle was deflected anteriorly [mean distance changes: posterior = 116.1% (SD ± 21.9%) vs. anterior = 100.0% (SD ± 0.0%), *p* = 0.008]. In line with the trapezoid ligament, the distance of the insertion of the AC capsule was significantly smaller when the inferior angle of the scapula was pointing laterally [mean length changes: medial = 118.0% (SD 18.8%) vs. lateral = 105.9% (SD ± 25.1%), *p* = 0.034].

## Discussion

The most important finding of this study is the significant impact of the scapular deflection on the varying distances of the coracoclavicular and acromioclavicular ligamentous insertions. According to the initial hypothesis, the ligamentous insertions were closer when the inferior angle pointed in the anterior direction. This is consistent with a situation where the patient’s shoulder is not ‘free-floating’ but is rather dorsally supported on the operating table.

As most anatomic and biomechanical studies regarding the acromioclavicular and coracoclavicular ligaments focused on the ligamentous length, insertion area as well as the different load distributions in a certain static position [2, 8], this study focused on the variation of distances between ligamentous insertions depending on the scapular position. To obtain a descriptive representation, the variations in distances were related to the maximum anterior deflection of the scapula.

Recently, Tuecking et al. demonstrated in a histology study, that the AC ligaments have an intrinsic ligamentous healing potential [20]. This healing potential may potentially be exploited when the required distance for healing is as short as possible, meaning that the ligamentous insertions are as close to each other as possible. In this biomechanical study, it was the case when the inferior angle pointed in the anterior direction. Shorter distances were only reached for the trapezoid in a situation with lateral scapular deflection. In the clinical routine, most of the shoulder arthroscopies are performed in the beach chair position, often with a ‘free floating’ shoulder, which results in a rather posterior deflection of the scapula and a larger distance between ligamentous insertions. This could be a potential explanation for the frequently observed loss of reduction in the follow-up [4].

In a biomechanical study, Oki et al. evaluated the contribution of the different AC joint ligaments concerning the range of motion of the scapula and clavicle in relation to the thorax in a dynamic model [15]. In a whole-body study, they investigated the different movement extensions based on sequential ligament cutting. For this purpose, the specimen’s arm was manually abducted and moved in the coronal and sagittal planes. They postulated that with intact ligaments, an elevation movement in the sagittal plane causes the scapula to tilt around the coracoclavicular attachments. This leads to a posterior rotation of the clavicle. However, as the centre of rotation of the scapula lies in its superior part, bony parts superior to this centre move posteriorly, but inferior parts such as the inferior angle experience a shift in an anterior direction. As this scapular posterior tilting decreased with sectioned coracoclavicular ligaments, the anterior deflection may be predetermined by intact, tensed ligaments. To rebuild this physiological state, it may be necessary to even tension the reconstructed ligaments with the scapula in anterior deflection. As proven in this study, a posterior deflection leads to nearly doubled distances between the ligamentous insertions. Reconstruction in this state may not be sufficient to build up enough tension during physiological movements and to rebuild the physiological tilt of the scapula demonstrated by Oki et al. [15].

Some limitations must be taken into account regarding the results of this study. In our test set-up, the surrounding soft tissues needed to be dissected to reach the ligamentous insertion. Furthermore, the movement of the scapula was guided artificially and no weight was applied to the joints. Due to a rather unrestricted movement into the maximum deflection, extreme values for the varying distances were obtained that may not be directly reproducible in a clinical situation. However, the results of this study are important to reconsider the patients positioning during tensioning of ligamentous reconstruction. A further limitation is the mean age of the investigated specimens that was considerably higher than the normal patient population affected by AC joint instability. Lastly, it must be analysed if this biomechanical effect will also be reflected in a clinical study when the scapular position with the inferior angle pointing anterior and medial is considered.

## Conclusion

Dorsal support shifting the inferior angle of the scapula anteriorly reduces the distance between the ligamentous insertions. Therefore, a patient position on a shoulder table with posterior support of the scapula is recommended to reliability reduce the acromioclavicular joint.

## Abbreviations

3D: Three-dimensional
AC: Acromioclavicular
CC: Coracoclavicular
DASH: Disabilities of arm, shoulder and hand
PU: Polyurethan
A: Acromion
SS: Scapula spine
SB: Scapula body
C: Clavicle
R: Romer arm
